# Genetic and morphological characterization of a new genotype of nervous necrosis virus circulating among Nile tilapia in the south of Egypt

**DOI:** 10.1007/s10123-023-00406-5

**Published:** 2023-07-29

**Authors:** Youssuf Ahmed Gherbawy, Maha Aboelkassem Thabet, Serageldeen Sultan

**Affiliations:** 1 Botany and Microbiology Department, Faculty of Science, South Valley University, Qena, 83523 Egypt; 2 Applied and Environmental Microbiology Center, South Valley University, Qena, 83523 Egypt; 3 Department of Microbiology, Virology Division, Faculty of Veterinary Medicine, South Valley University, Qena, 83523 Egypt

**Keywords:** Nervous necrosis virus, Nile tilapia, Phylogenetic analysis, New genotype, Egypt

## Abstract

Nervous necrosis virus (NNV) is the causative agent of viral nervous necrosis in freshwater and marine fishes. In this study, NNV circulating among wild and farmed Nile tilapia (*Oreochromis niloticus*) was genetically and morphologically characterized using reverse transcription polymerase chain reaction (RT-PCR), sequencing analysis, and transmission electron microscopy (TEM). Brain, eye, and other organ (spleen, kidney, heart, and liver) specimens were collected from 87 wild (66) and farmed (21) Nile tilapia fish during their adult or juvenile stage at different localities in Qena and Sohag governorates in southern Egypt. Among them, 57/87 fish showed suspected NNV clinical signs, and 30/87 were healthy. The results revealed that NNV was detected in 66 out of 87 fish (58.62% in the wild and 17.24% in farmed Nile tilapia by RT-PCR), and the prevalence was higher among diseased (55.17%) than in healthy (20.69%) fish. NNV was detected in the brain, eye, and other organs. Using TEM, virion size variations based on the infected organs were observed. Nucleotide sequence similarity indicated that NNVs had a divergence of 75% from other fish nodaviruses sequenced in Egypt and worldwide. Phylogenetic analysis distinguished them from other NNV genotypes, revealing the emergence of a new NNV genotype in southern Egypt. In conclusion, NNV is circulating among diseased and healthy Nile tilapia, and a new NNV genotype has emerged in southern Egypt.

## Introduction

Nervous necrosis virus (NNV) causes viral nervous necrosis (VNN) disease, also known as viral encephalopathy and retinopathy (VER), which is an acute and severe disease in fish caused by betanodavirus (Munday et al. 2002; Bandín and Souto 2020). NNV belongs to the genus betanodavirus in the family *Nodaviridae* and is an icosahedral, non-enveloped virus with a diameter of 25–30 nm (Johansen et al. 2004; Rajan et al. 2016; Bandín and Souto 2020). The NNV genome consists of two segments of positive single-stranded RNA. The large segment RNA1 (3.1 kb) encodes the RNA-dependent RNA polymerase (RdRp), and the small segment RNA2 (1.4 kb) encodes the coat protein (CP) (Doan et al. 2017; Volpe et al. 2023). In addition, a sub-genomic RNA, RNA3, is transcribed from RNA1 3′ end (Iwamoto et al. 2005; Sommerset et al. 2005). The RNA3 encodes B1 and B2, which are involved in immune evasion (Su et al. 2018).

Betanodaviruses have traditionally been classified into four genotypes based on a small variable sequence of RNA2, called the T4 region (Olveira et al. 2009; Toffan et al. 2017). The red-spotted grouper NNV (RGNNV) genotype, which affects warm-water species, is the most widely distributed genotype, with the highest number of susceptible species. The barfin founder NNV (BFNNV) genotype is limited to cold-water fishes. The tiger puffer NNV (TPNNV) genotype has been described in only one species in Japan. Striped jack NNV (SJNNV) has long been detected in the Iberian Peninsula (Cutrín et al. 2007). Three additional genotypes have been suggested, namely turbot nodavirus (TNV) (Johansen et al. 2004), Atlantic cod NNV (ACNNV) (Gagné et al. 2004), and the Korean shellfish NNV (KSNNV) (Kim et al. 2019). TNV has been widely established as genotype five NNV (Korsnes et al. 2017). The RGNNV genotype has been reported in Egypt, causing severe mortality in hatchery-reared juvenile fish (Taha et al. 2020).

NNV has been isolated from farmed and wild fish in different regions, including South and East Asia (China, Japan, Taipei, India, Iran, Indonesia, Korea, Thailand, Malaysia, Philippines, and Vietnam), Oceania (Tahiti and Australia), Mediterranean (France, Israel, Greece, Italy, Malta, Portugal, Spain, and Tunisia), the UK, Caribbean, Norway, North America (Canada and the USA) (Munday et al. 2002), and South Africa (Taha et al. 2020). NNV has been associated with more than 120 fish species worldwide (Costa and Thompson 2016), most of which are marine, and causes neuropathological conditions (Munday et al. 2002; Panzarin et al. 2012). In addition, it affects freshwater fishes (Chi et al. 2003; Bovo et al. 2011).

The target tissues affected by NNV are the brain, spinal cord, central nervous system (CNS), and retina (Munday et al. 2002; Bovo et al. 2011). Although NNV mainly affects small fish (larval and juvenile stages), severe mortalities of up to 100% have been reported for market size and adult fish (OIE 2019). Fish affected by NNV show clinical signs such as loss of appetite, skin darkening, loss of sight, and abnormal swimming behavior (spiral swimming, horizontal looping, whirling, darting, lying down at the tank bottom, or swimming rapidly in circles or straight-ahead). The swim bladder hyperinflation, coloration abnormalities (pale or dark), lesions on the body and fins, backbone deviation, rotten, fins, and abdominal swelling were recorded (Bandín and Souto 2020; Toubanaki et al. 2022). These clinical signs depend on the biological stage, fish species, disease stage, and temperature (Bandín and Souto 2020). Histopathological analyses include necrosis, neuronal degeneration, and vacuolation of the retina (Tanaka et al. 2004). NNV can be transmitted using horizontal and vertical methods (OIE 2019). Horizontal transmission is considered the most common disease spreading from fish to fish or contaminated water (Gomez et al. 2010; Kang et al. 2023). Globally, this virus causes severe economic losses in diverse marine and freshwater fish species (Bandín and Souto 2020). NNV control is difficult owing to the high stability of NNV particles in the environment (Frerichs et al. 2000; Adachi et al. 2007).

The development of molecular techniques, such as reverse transcription polymerase chain reaction (RT-PCR) targeting a portion of the coat protein gene (RNA2) of betanodavirus, has been considered an efficient diagnostic tool for the identification of NNV (Dalla Valle et al. 2005). Although NNV outbreaks have been reported worldwide, to the best of our knowledge, only one report has been published concerning the detection of NNV in Egypt (Taha et al. 2020). They have identified NNV from collected samples from three tilapia hatcheries and brood stocks showing high mortalities located in Kafr Elsheikh and El Beheira in the north of Egypt in 2018 and 2019 using RT-PCR and transmission electron microscopy (TEM). The obtained NNV nucleotide sequences were phylogenetically related to the RGNNV genotype with 97.2–98.3% nucleotide identity. The virion particles have an average size of 38.2–53.5 nm. Also, NNV nucleic acid could be detected in experimentally infected tilapia using the prepared tissue homogenates from collected samples during outbreaks among fish (Taha et al. 2020). Therefore, the current study was performed to detect NNV by RT-PCR using specific primers targeting the RNA2 segment in clinical and subclinical infected specimens collected from farmed and wild tilapia (*Oreochromis niloticus*) fish and to characterize the morphological and genetic features of NNV by TEM and phylogenetic analysis of RNA2 sequences, respectively, in southern Egypt.

## Materials and methods

### Collection and preparation of fish samples

A total of 87 diseased (57) and healthy (30) fish samples of Nile tilapia were randomly collected from different markets (66 wild fish) and farms (21 farmed fish) at different localities in Sohag and Qena governorates in southern Egypt, from September 2019 to November 2020. The fish were in the juvenile and adult stages. The samples were immediately transferred to the virology laboratory of the Faculty of Veterinary Medicine, South Valley University, in an icebox. The organs (brain, eye, kidney, spleen, heart, and liver) from each fish were individually collected and stored at −80 °C until use. The data of the collected fish, including the number of fish, year of collection, localities, source, culture system, age, weight, and clinical signs, were recorded (Table 1).

**Table 1:** Information about the collected fish samples in this study

Source (locality)	Fish number	Type (origin)	Stage*	Weight in gram	Year	Clinical signs
Market (Alsharij-Qena)	8	Wild (Lake Nasser)	Adult	300–400	2019	Absent
Market (Alsharij-Qena)	2	Farm (Kafr Elsheikh)	Adult	300–400	2019	Absent
Market (Alsharij-Qena)	9	Wild (Lake Nasser)	Adult	150–200	2019	Present
Market (Alsharij-Qena)	2	Farm (Kafr Elsheikh)	Adult	300–400	2019	Present
Market (Alqasaria-Sohag)	7	Farm (Kafr Elsheikh)	Adult	300–500	2019	Absent
Market (Alqasaria-Sohag)	2	Wild (Lake Nasser)	Adult	200	2019	Present
Farm (Qeft-Qena)	3	Farm (Qeft)	Juvenile	10–20	2019	Present
Market (Alsharij-Qena)	7	Wild (Lake Nasser)	Adult	120–150	2020	Present
Market (Alsharij-Qena)	19	Wild (Lake Nasser)	Adult	100–120	2020	Present
Market (Alsharij-Qena)	15	Wild (Lake Nasser)	Adult	100–120	2020	Present
The Nile River (Dandrah-Qena)	6	Wild (Nile River)	Juvenile	10–20	2020	Absent
Market (Aluminum-Qena)	7	Farm (Kafr Elsheikh)	Adult	500	2020	Absent

*Juvenile = 10–25 g; adult > 25 g

### Reverse transcription polymerase chain reaction (RT-PCR)

According to the manufacturer’s instructions, the total RNA was extracted from the specimens using the RNeasy Mini Kit (Qiagen, Germany). The extracted RNA was used to detect the presence of NNV by RT-PCR using EasyScript® one-step RT-PCR Super Mix (Transgen Biotech, China) according to the manufacturer’s instructions and primers targeting the RNA2 gene, VNNF:5′-ACA CTG GAG TTT GAA ATT CA-3′ and VNNR:5′-GTC TTG TTG AAG TTG TCC CA-3′, which amplified 605 bp (Dalla Valle et al. 2000). In brief, a total reaction volume of 20 μl contains 10 μl of 2 × ES One-Step Reaction Mix, 0.4 μl of EasyScript One-Step Enzyme Mix, 0.4 μl of each primer (20 pmol), 3.8 μl of RNase-free water, and 5 μl of template RNA. The optimized thermal cycling conditions for one-step RT-PCR were as follows: a cDNA synthesis step at 45 °C for 30 min, PCR amplification as an initial denaturation cycle at 94 °C for 5 min, and 35 cycles of denaturation at 94 °C for 30 s, annealing at 49 °C for 30 s, and extension at 72 °C for 1 min. The final extension step was performed at 72 °C for 10 min. The PCR products were analyzed by 1.5% agarose gel electrophoresis and stained with ethidium bromide at 0.5 μg/ml concentration.

### Transmission electron microscope (TEM) for morphological characterization

Brain, eye, and spleen tissue samples of suspected diseased tilapia fishes were collected and kept in 2.5% glutaraldehyde (Sigma Aldrich, St. Louis, MO, USA) in phosphate-buffered saline (PBS; 0.1 mol l–1, pH 7.4) for TEM, which was carried out at the Transmission Electron Microscopy Laboratory, Faculty of Agriculture, Cairo University Research Park. The tissue samples were sliced into tiny slices (1 mm) and fixed for 2 h in 2.5% glutaraldehyde (Sigma Aldrich, St. Louis, MO, USA) in PBS at 4 °C. Then, put in 1% osmium tetroxide (Sigma Aldrich, St. Louis, MO, USA) in PBS for 1 h at 4 °C and dehydrate in alcohol. Microtome sections were created using a Leica Ultra Cut microtome (UCT) at a thickness of approximately 500–1000 μm. Toluidine blue (×1) (Sigma) was used to stain thin sections, which were analyzed using a Leica ICC50 HD camera, whereas uranyl acetate and lead citrate were used to stain ultrathin slices that were almost 75–90 μm thick. Subsequently, a TEM JEOL (JEM-1400 TEM) was used to analyze the samples. Images were captured using a CCD camera model AMT and an optronic camera with a 1632 × 1632 pixel format as the side-mount configuration. The camera had a 1394 firewire board for acquisition.

### Nucleotide sequencing analysis

The obtained PCR product (605 bp) was sequenced for four NNV-positive specimens from different organs using the QIAquick PCR Purification Kit (QIAGEN, Valencia, CA, USA) for PCR product purification. A BigDye Terminator ver. 3.1 Cycle Sequencing Kit (Applied Biosystems, Foster City, CA, USA) was used for the sequence reaction of the purified PCR products. Sanger sequencing was conducted at Macrogen, Korea, using an ABI PRISM 3730XL analyzer (96 capillary types). Nucleotide sequencing was performed by using the primer sets described above. The bio-edit package ver. 7.2 software (http://www.mbio.ncsu.edu/BioEdit/bioedit.html) was used to analyze the obtained sequence data compared to other sequences retrieved from GenBank by a BLAST homology search (http://www.ncbi.nlm.nih.gov/genomes/FLU/FLU.html). The nucleotide alignments of the selected sample sequences were compared with those of other NNV genotype sequences.

### Phylogenetic analysis

The nucleotide sequences of NNV were aligned with other betanodavirus isolates deposited in GenBank, which represent various NNV genotypes worldwide. Gaps and missing data were removed from all the positions. MEGA 6.06 software created a phylogenetic tree for the partial RNA2 gene encoding the coat protein using the general time reversible model in maximum likelihood (ML) with 1000 bootstrap replicates (Tamura et al. 2013).

## Results

### Gross lesions and clinical observations

**Fig. 1:**
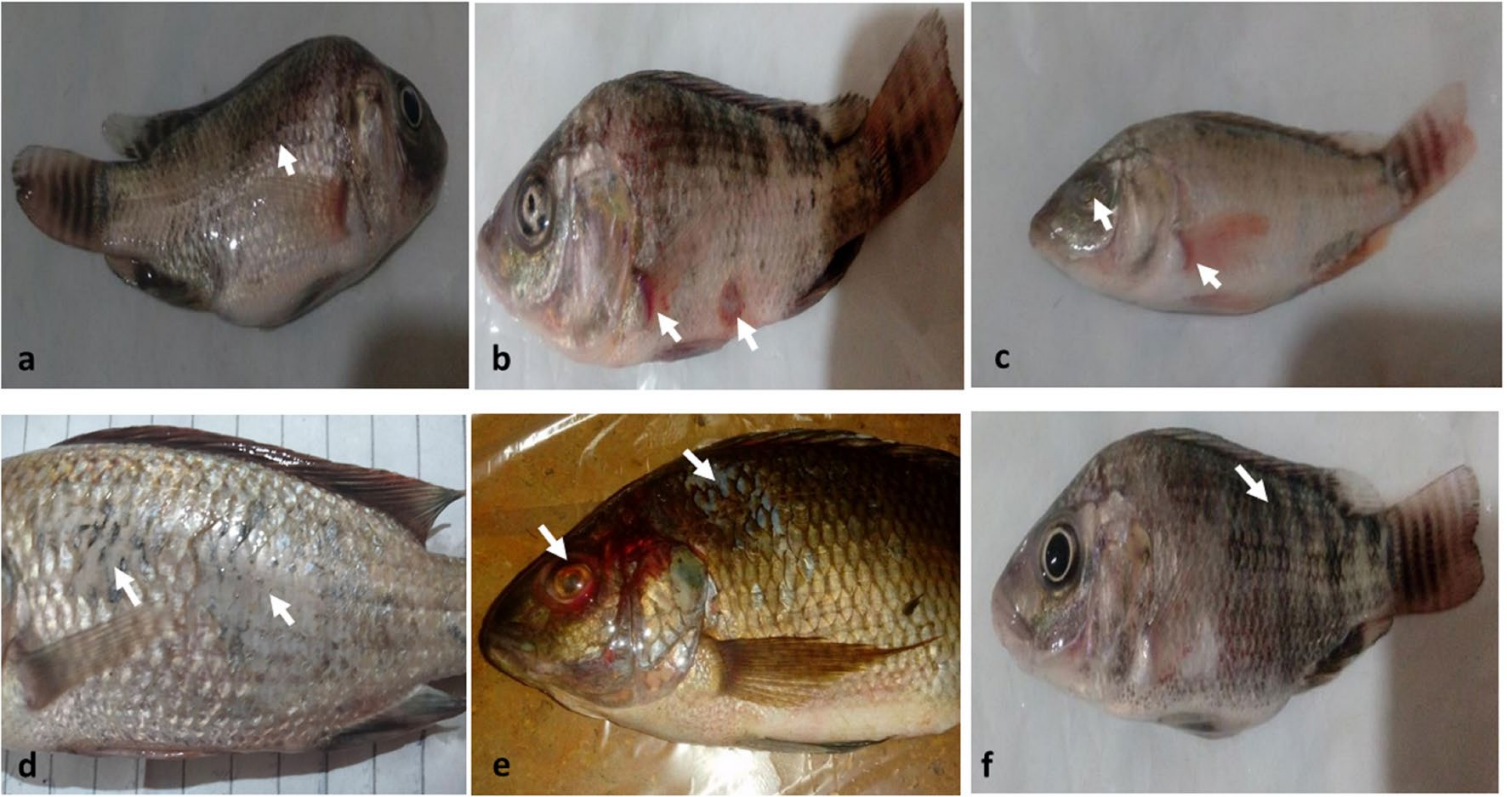
The clinical signs of NNV were observed on tilapia fish: **a**) abdominal swelling, **b**) hemorrhagic patches, **c**) exophthalmia and hemorrhage at the base of the pectoral fin, **d**) detached scales, **e**) redness of the eye and head, and detachment of scales, and **f**) skin darkening

The diseased fish showed gross lesions of skin darkening, detached scales, hemorrhagic patches, exophthalmia, abdominal swelling, weight loss, and organ redness (Fig. 1).

### Detection of NNV in fish samples by RT-PCR

**Fig. 2:**
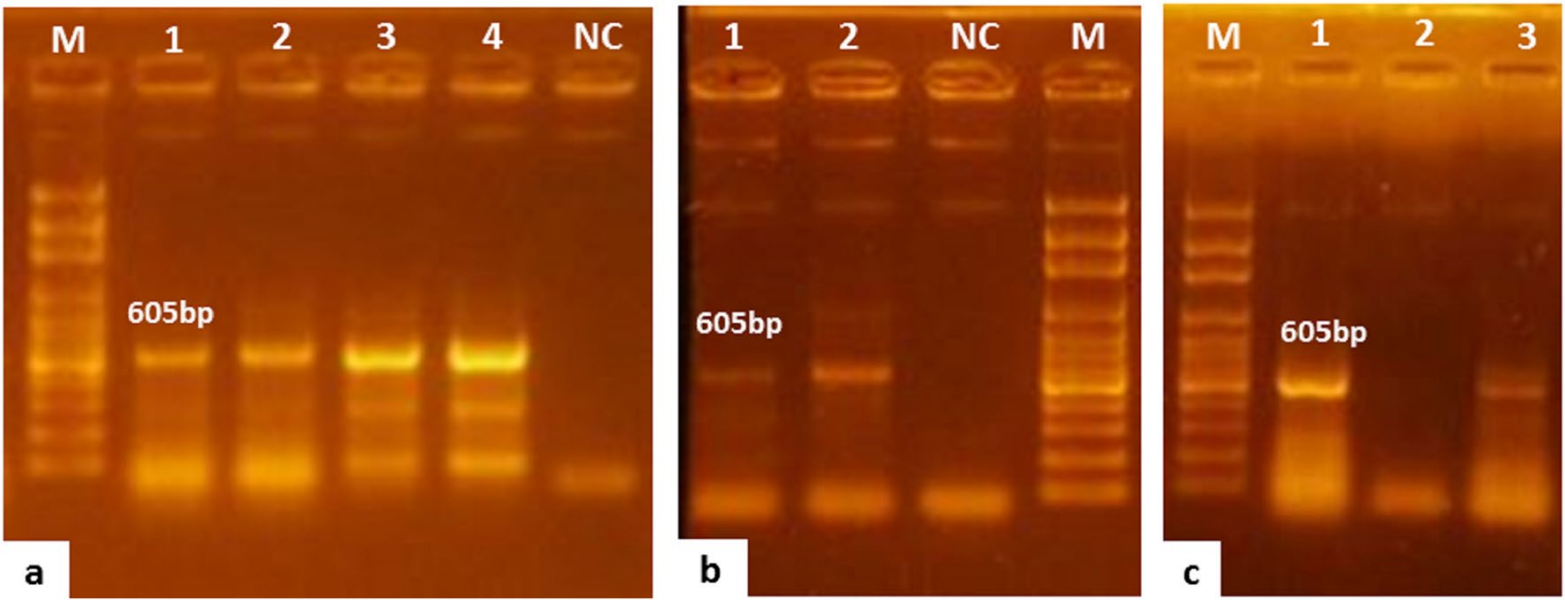
RT-PCR detection of NNV in different specimens using primers targeting 605 bp of the RNA2 gene; **a**) lanes 1 and 2: positive NNV from kidney specimen; lanes 3 and 4: positive NNV from spleen specimen; M:100 bp DNA ladder; NC, negative control. **b**) Lane 1: positive NNV from eye specimen; lane 2: positive NNV from brain specimen; M:100 bp DNA ladder; NC, negative control. **c**) Lanes 1 and 3: positive NNV from heart specimen; lane 2: negative NNV from liver specimen; M:100 bp DNA ladder

A total of 66 out of 87 fish were positive for NNV by RT-PCR, producing 605 bp (Fig. 2). Of them, 15 samples were farmed fish from Kafr Elsheikh and Qeft hatcheries, and 51 were wild fishes from Lake Nasser and the Nile River (Table 2). The NNV-positive samples were higher in diseased (48/57) than in healthy (18/30) Nile tilapia (Table 2). All samples collected from juvenile fish (9/9) had NNV, while most adult fish (57/78) were virus-positive (Table 2). The prevalence of NNV was higher in the eye (3/3), heart (6/6), kidneys (21/24), and brain (30/36) than in the spleen (6/15). No NNV was detected in liver samples, as shown in Table 2 and Fig. 2.

**Table 2:** Characteristic features of RT-PCR NNV positive fishes collected at different regions

Characteristics	Sample number	NNV	Percentage (%)
RT-PCR			
Positive	87	66	75.86
Negative		21	24.14
Age			
Adult	78	57	65.52
Juvenile	9	9	10.34
Type of fish			
Wild	66	51	58.62
Farm	21	15	17.24
Clinical signs			
Apparently health	30	18	20.69
Diseased	57	48	55.17
Organs			
Brain	36	30	34.48
Eye	3	3	3.45
Spleen	15	6	6.89
Kidney	24	21	24.14
Heart	6	6	6.89
Liver	3	0	0
Governorate			
Qena	78	60	68.97
Sohag	9	6	6.89

### Morphological characterization of NNV in different organs by TEM

**Fig. 3:**
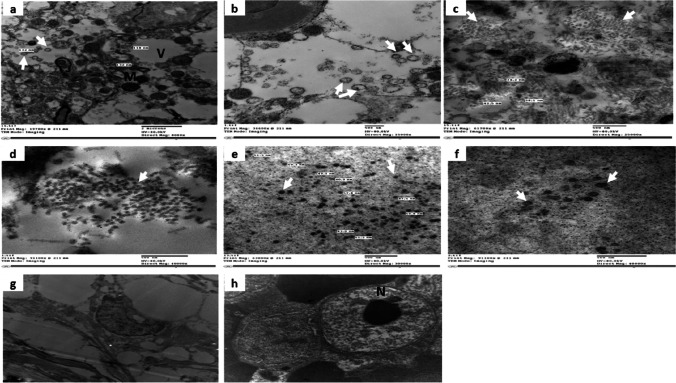
TEM of NNV occurrence in different organs (brain, eye, and spleen) of Nile tilapia. **a**) Non-enveloped and icosahedral viral particles in brain tissues arranged randomly in the cytoplasm (arrow), intracytoplasmic vacuoles (V) in brain tissues, size range 98–132 nm (mag: ×8000, bar = 200 nm). **b**) Viral particles in brain tissue (arrow) (mag: ×15,000, bar = 500 nm). **c**) Viral particles as clusters in eye tissues (arrows), size ranged 26.9–54.5 nm (mag: ×25,000, bar = 500 nm). **d**) Viral particles in eye tissues (arrow) (mag: ×40,000, bar = 500 nm). **e**) Viral particles in spleen tissues arranged randomly in the cytoplasm (arrow), size ranging from 37.9 to 63.9 nm (mag: ×30,000, bar = 500 nm). **f**) Viral particles (arrows) (mag: ×40,000, bar = 500 nm). **g**) Negative control brain tissue (mag: ×8000, bar = 200 nm). **h**) Negative control spleen tissue (mag: ×10,000, bar = 500 nm). M, mitochondria; V, vacuole; N, nucleolus

TEM examination of eye, brain, and spleen specimens from diseased fish, as well as healthy fish as a negative control, showed many virus particles arranged randomly in the cytoplasm or in groups in the form of arrays with non-enveloped icosahedral symmetry. Variations in viral particle sizes were observed, where the size in the brain was 98–132 nm, in the eye was 26.9–54.5 nm, and in the spleen was 37.9–63.9 nm, as shown in Fig. 3a–f. In addition, the infected tissues showed intracytoplasmic vacuoles (Fig. 3a). No viral particles were present in the negative control fish specimens (Fig. 3g, h).

### Sequencing and phylogenetic analysis

**Fig. 4:**
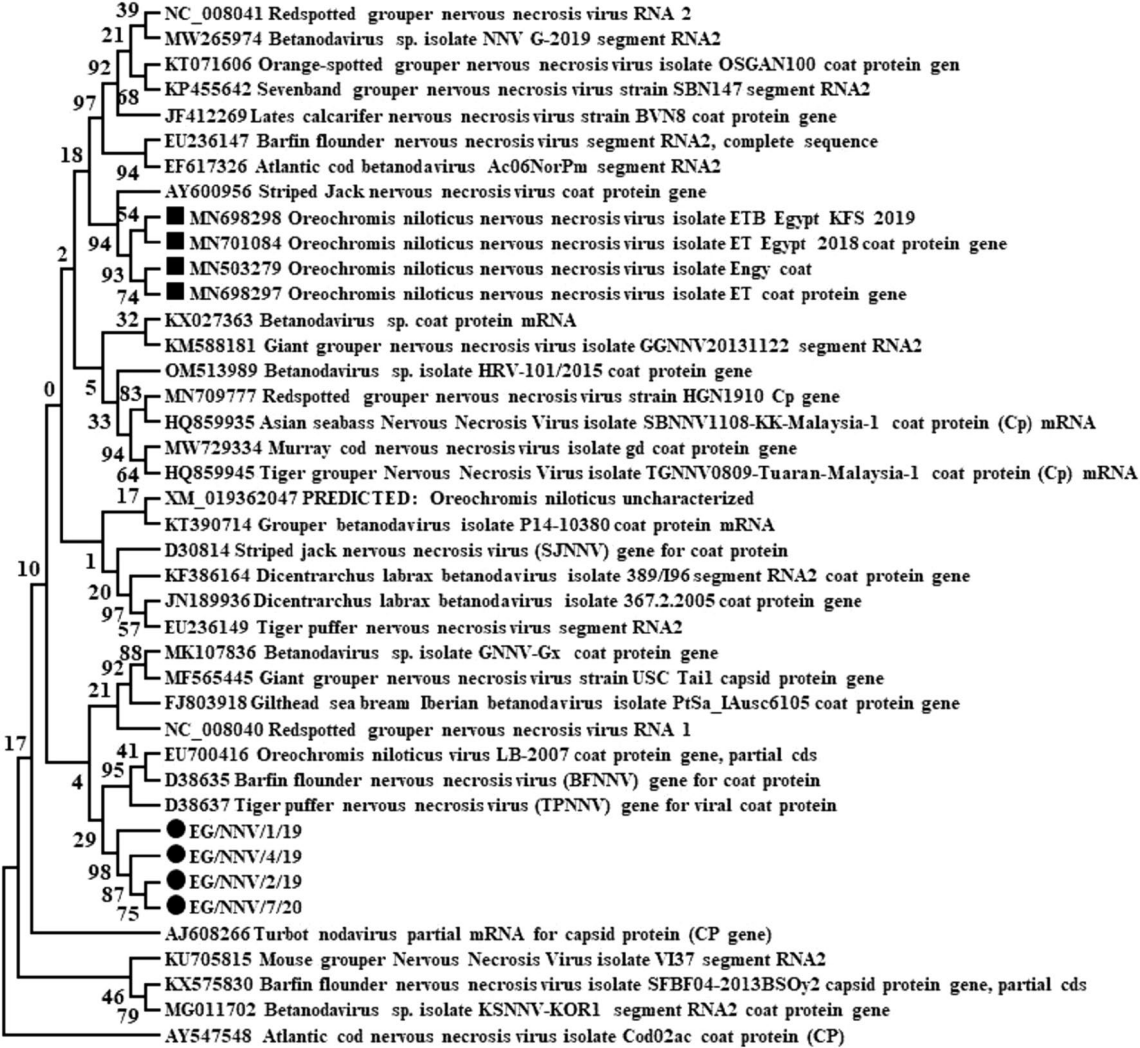
Molecular phylogenetic tree of partial RNA2 gene sequences using the maximum likelihood method based on the general time-reversible model. The analysis involved 41 sequences, and all positions containing gaps and missing data were eliminated. The final dataset contained 405 positions. Evolutionary analyses were conducted using MEGA 6 software, and the NNV sequences of this study are indicated by a circle (●), whereas Egyptian isolates are indicated by a square (■)

**Fig. 5:**
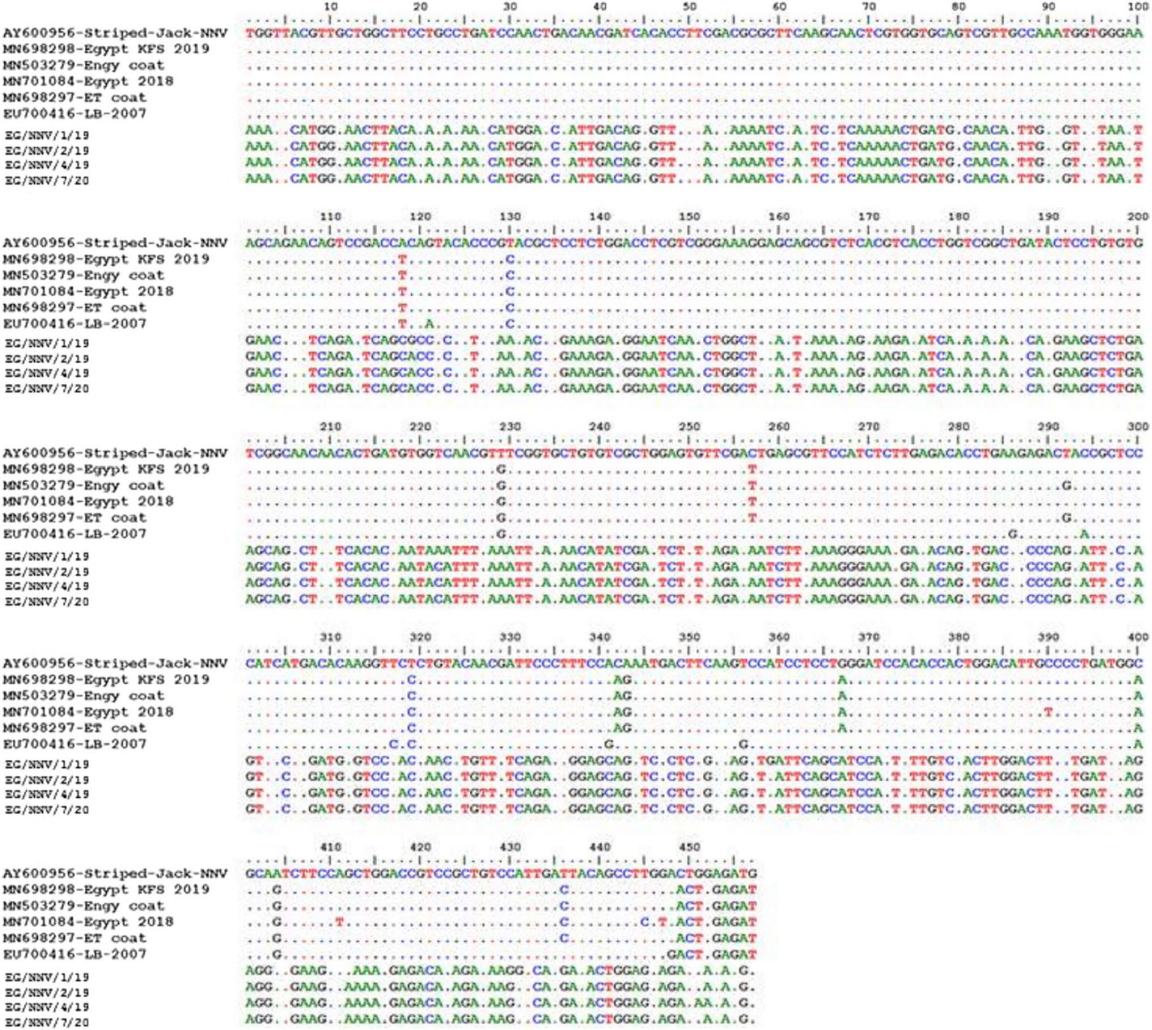
Multiple nucleotide sequences alignment of the partial RNA2 gene encoding the coat protein of four NNV-positive specimens from Tilapia fish in this study and other betanodavirus genotypes: *Oreochromis niloticus* nervous necrosis virus (RGNNV, MN698298, MN701084, MN503279, and MN698297), an Egyptian isolate; striped jack nervous necrosis virus (SJNNV, AY600956), a Greece isolate; and *Oreochromis niloticus* nervous necrosis virus (RGNNV, EU700416), an European isolate

A phylogenetic analysis of the four NNV-positive specimens from different organs, years of collection, stages of growth, and clinical signs (Table 3) of the partial RNA2 gene was conducted. The results indicated that all 4 NNV-positive specimens did not cluster with any Egyptian NNV or other genotypes of betanodaviruses retrieved from GenBank (Table 4) and formed a single cluster with a new genotype of NNV. This was related to the EU700416 *Oreochromis niloticus* virus and away from Egyptian NNV isolates (Fig. 4). In addition, the four NNV-positive specimen sequences in this study showed identities ranging between 98.6 and 100% (Table 5). Nucleotide alignment of four NNV-positive specimen sequences showed similarity to other Egyptian NNV isolates from El Beheira Fries and Kafr Elsheikh (MN698298, MN503279, MN701084, and MN698297) of 24.7–25.1%. It is similar to the EU700416 *Oreochromis niloticus* virus with 24.2–24.5% identity and to the AY600956 striped jack nervous necrosis virus with 24.7–24.9% identity, as shown in Table 5 and Fig. 5. The high divergence rates among the four NNV-positive specimens and other Egyptian isolates, EU700416 *Oreochromis niloticus* virus and AY600956 striped jack nervous necrosis virus, were 75.3–74.9%, 75.8–75.5%, and 75.3–75.1%, respectively, as shown in Table 5 and Fig. 5.

**Table 3:** Characters of sequenced 4 NNV obtained from fish samples

ID	Type (origin)	Stage*	Organ	Weight in gram	Year	Clinical signs
EG/NNV/1/19 (OR290787)^**^	Farm-Sohag (Kafr Elsheikh)	Adult	Kidney	300	2019	Absent
EG/NNV/2/19 (OR290788)	Wild-Qena (Lake Nasser)	Adult	Heart	150	2019	Detached scales, skin darkening, and hemorrhage
EG/NNV/4/19 (OR290786)	Farm-Qena (Qeft)	Juvenile	Spleen	10–20	2019	Abdominal swelling, hemorrhagic patches, exophthalmia, detached scales, skin darkening, and high mortality rate
EG/NNV/7/20 (OR290785)	Wild-Qena (Lake Nasser)	Adult	Eye	100	2020	Detached of scales, redness in the eye, and skin darkening

*Juvenile = 10–25 g; adult > 25 g ** Assigned Genbank accession numbers for NNV sequences of the current study

**Table 4:** Retrieved nucleotide sequences of different NNV genotypes from GenBank for phylogenetic analysis

Accession number	Genotype	Host	Country
HQ859945	RGNNV	Brown-marbled grouper (*Epinephelus fuscoguttatus*)	Malaysia
HQ859935	RGNNV	Asian seabass (*Lates calcarifer*)	Malaysia
MN709777	RGNNV	Hybrid grouper (*Epinephelus fuscoguttatus* x *E. lanceolatus*)	China
OM513989	RGNNV	Mullet (*Chelon labrosus*)	Croatia
KM588181	RGNNV	Giant grouper (*Epinephelus lanceolatus*)	Taiwan
EU236147	BFNNV	Barfin flounder	Japan
KF386164	RGNNV	Sea bass (*Dicentrarchus labrax*)	Italy
JF412269	RGNNV	Asian seabass (*Lates calcarifer*)	India
KP455642	RGNNV	Sea perch (*Lateolabrax japonicus*)	China
KT071606	RGNNV	Orange-spotted grouper (*Epinephelus coioides*)	Taiwan
EU700416	RGNNV	Tilapia (*Oreochromis niloticus*)	Europe
KT390714	RGNNV	Giant grouper (*Epinephelus lanceolatus*)	Australia
NC_008040	RGNNV	Unknown	Unknown
MN698298	RGNNV	Tilapia (*Oreochromis niloticus*)	Egypt
MN701084	RGNNV	Tilapia (*Oreochromis niloticus*)	Egypt
MN503279	RGNNV	Tilapia (*Oreochromis niloticus*)	Egypt
MN698297	RGNNV	Tilapia (*Oreochromis niloticus*)	Egypt
AJ608266	TNV	Turbot (*Scophthalmus maximus*)	Norway
AY547548	ACNNV	Atlantic cod	Canada
MG011702	KSNNV	*Crassostrea gigas*	South Korea
EF617326	BFNNV	Atlantic cod	Norway
D38635	BFNNV	Barfin flounder	Japan
D30814	SJNNV	Striped jack	Japan
JN189936	SJNNV	Sea bass (*Dicentrarchus labrax*)	Italy
FJ803918	SJNNV	Gilthead sea bream	Portugal
D38637	TPNNV	Tiger puffer	Japan
EU236149	TPNNV	Tiger puffer	Japan
MW729334	Unpublished	Murray cod (*Maccullochella peelii*)	Unknown
NC_008041	Unpublished	Unknown	Unknown
MW265974	Unpublished	Hybrid grouper (*Epinephelus lanceolatus* x *Epinephelus fuscoguttatus*)	Thailand
XM_019362047	Chromosome	Tilapia mRNA	
AY600956	Unpublished	Striped Jack	Greece
Ku705815	Unpublished	Humpback grouper (*Cromileptes altivelis*)	Vietnam
KX575830	Unpublished	Oyster (*Crassostrea gigas*)	South Korea
KX027363	Unpublished	Hybrid grouper (*Epinephelus fuscoguttatus* x *Epinephelus lanceolatus*)	China
MK107836	Unpublished	Hybrid grouper (*Epinephelus fuscoguttatus* x *Epinephelus lanceolatus*)	China
MF565445	Unpublished	Giant x tiger grouper hybrid	Taiwan

**Table 5:** Nucleotide sequence identities of 4 NNV positive specimens in this study and other Egyptian, European, and Greek betanodaviruses

ID	1	2	3	4	5	6	7	8	9	10
1- EG/NNV/1/19	ID	98.9	98.6	98.9	24.7	24.9	24.7	24.9	24.2	24.7
2- EG/NNV/2/19	98.9	ID	99.7	100	24.9	25.1	24.9	25.1	24.5	24.9
3- EG/NNV/4/19	98.6	99.7	ID	99.7	24.9	25.1	24.9	25.1	24.5	24.7
4- EG/NNV/7/20	98.9	100	99.7	ID	24.9	25.1	24.9	25.1	24.5	24.9
5- MN698298 NNVETB Egypt KFS 2019	24.7	24.9	24.9	24.9	ID	99.7	99.1	99.7	97.3	95.8
6- MN503279 NNV Engy coat	24.9	25.1	25.1	25.1	99.7	ID	98.9	100	97.1	95.6
7- MN701084 NNVET Egypt 2018	24.7	24.9	24.9	24.9	99.1	98.9	ID	98.9	96.4	94.9
8- MN698297 NNV ET	24.9	25.1	25.1	25.1	99.7	100	98.9	ID	97.1	95.6
9- EU700416 NNV LB-2007^a^	24.2	24.5	24.5	24.5	97.8	97.1	96.4	97.1	ID	95.4
10- AY600956 Striped Jack NNV^b^	24.7	24.9	24.7	24.9	95.8	95.6	94.6	95.6	95.4	ID

^a^NNV genotype RGNNV isolated from Tilapia (*Oreochromis niloticus*) in Europe

^b^NNV isolated from striped jack in Greece

## Discussion

NNV causes massive mortality and significant economic losses in cultured marine and freshwater fish species worldwide (Bovo et al. 2011; Bandín and Souto 2020). Two RNA viruses have recently been identified as the leading causes of increased tilapia mortality (90–100%), tilapia lake virus (TiLV) and NNV (Pulido et al. 2019). Tilapia farms and hatcheries in Egypt have suffered from mass mortality (up to 70%) in the past few years owing to NNV (Taha et al. 2020). There is little information on the effect of NNV on Egyptian aquatic environments, especially tilapia. To our knowledge, this is the second report of NNV among tilapia in Egypt. Therefore, this study investigated the occurrence of NNV in subclinical, clinical, adult, and juvenile tilapia fish in the south of Egypt. Several clinical signs related to NNV were observed in examined fish, including skin darkening, abdominal swelling, high mortality rate (up to 70%), detached scales, hemorrhagic patches, exophthalmia and redness of organs, and exophthalmia and retina lesions; this is in agreement with Zorriehzahra et al. (2014), Toffan et al. (2016), Ariff et al. (2019), and Taha et al. (2020). RT-PCR results revealed that 66 out of 87 (75.9%) specimens were positive for NNV. The results obtained in this study are lower than those reported by Taha et al. (2020), who found NNV in all the RNA extracted from fry pools in Egypt. This difference may be due to fish size (juvenile or adult), environmental conditions (farm or natural), and fish status (diseased or apparently health). NNV was detected in farmed and wild tilapia, adult and juvenile stages, and subclinically and clinically infected fish. This is attributed to the fact that water and freshwater can horizontally transmit NNV. If the virus is present in live fish, they can become carriers of the virus without the appearance of any clinical signs (Gomez et al. 2008). The presence of NNV in water plays a vital role in transmitting viruses to all healthy fish, whether cultured, wild, juvenile, or adult (Gomez et al. 2008). A previous study proposed that NNV transmission via vertical (Valero et al. 2015) or horizontal transmission may occur because of asymptomatic NNV carriers, virus-contaminated food, and poor biosecurity measures (Hick et al. 2011).

NNV appeared to have a more significant effect on larger and adult fish than on smaller and younger (larval and juvenile) fish (Ariff et al. 2019). Age is a primary risk factor for susceptibility to NNV (Hick et al. 2011). In this study, NNV was detected in the brain, eye, and other organs (spleen, kidney, and heart) but not in the liver, indicating that the virus caused a systemic infection. Lopez-Jimena et al. (2012) reported the presence of nodavirus in European seabass’s internal organs (spleen, kidney, and liver). The presence of viral proteins in these organs does not mean that they are active in virus replication, as viral proteins may have been carried there as immune complexes by host defense mechanisms (Húsgağ et al. 2001). Infection of Egyptian tilapia with NNV has been detected in adult and juvenile stages in the brain, eye, spleen, kidney, and heart of subclinical and clinical fish samples collected from wild and farmed tilapia in the current study. In Japan, high frequencies of NNV (67%) have been found in healthy cultured and wild marine fishes (Gomez et al. 2004). Viral particles were also detected in the spleen, heart, and kidney specimens collected from the fish in the current study. The retina and central nervous system, including the brain and spinal cord, are the target organs of the NNV (Bovo et al. 2011). The kidney, spleen, and heart are not considered target organs and are therefore unsuitable for NNV diagnosis; however, the causative agent of the disease can be detected in many organs (Sitar et al. 2021). Our results revealed that the kidney, heart, and spleen tissues could be suitable for virus analysis, in addition to the eye and brain (Sitar et al. 2021). NNV can remain infectious at various temperatures (Binesh and Greeshma 2013). Environmental conditions should also be considered as a source of nodavirus transmission to marine fishes (Gomez et al. 2008). According to an early study, betanodavirus genotypes are not strongly associated with specific host species but rather with geographic region and water temperature (cold to warm) (OIE 2019).

Non-enveloped and spherical shapes with icosahedral symmetry virus particles were also observed in the infected brain, eye, and spleen using TEM. The average size of the NNV particles in infected tilapia tissues was more considerable than in the previous study, which reported that the size of viral particles ranged from 25 to 33 nm (Sahul Hameed et al. 2019). The current NNV particles detected by TEM were similar to other Egyptian virus particles (Taha et al. 2020) only in eye and spleen tissues, but the brain tissue had bigger virus particles than other Egyptian isolates. This size variation indicated the presence of mutations in another place in the genome that affect the number of capsomeres and/or 3D structure (Taha et al. 2020) or may be due to the difference in environmental conditions, especially temperature between the north and the south of Egypt or the strain of the virus. Infected tissues show intracytoplasmic vacuoles (Zorriehzahra et al. 2014). Also, virus particles are arranged randomly in the cytoplasm (Maeno et al. 2004) or in groups in the form of arrays similar to Taha et al. (2020).

Nucleotide sequence alignment of NNV-positive specimens with other betanodaviruses revealed lower sequence identities of 24.2–25.1% and higher dissimilarities of 74.9–75.8%; also, phylogenetic analysis indicated that these NNV sequences could not be placed within the four major established genotypes or the other three suggested genotypes of fish nodaviruses. This high dissimilarity between the current NNV sequences and betanodavirus genotype sequences indicated that these NNV sequences were considered a new genotype of betanodaviruses. This high dissimilarity between the present NNV sequences and other Egyptian virus sequences may be due to differences in environmental conditions in the south of Egypt or genetic variations. The detection of a new NNV genotype in tilapia in the south of Egypt coincides with the fish farming industry, indicating the need for further investigation and continuous surveillance of this new genotype. The question of whether a new genotype of NNV poses a threat of cross-infection with other wild or farmed species in Egypt is particularly crucial to answer.

## Conclusion

The present study revealed that NNV circulates among tilapia fishes, either healthy or diseased, and could be detected in different growth stages and organs. Also, the results showed that a new betanodavirus genotype with varying particles of virion size based on the infected organs had been detected and was circulating among farmed and wild tilapia fishes in freshwater environments in the south of Egypt.

## Data Availability

The authors confirmed that the data supporting the finding of this study are available in the article.
